# Thematic series CAPMH “Forensic Child and Adolescent Psychiatry and Mental Health”

**DOI:** 10.1186/s13034-016-0126-3

**Published:** 2016-11-01

**Authors:** Klaus Schmeck, Cyril Boonmann, Jörg M. Fegert

**Affiliations:** 1 Child and Adolescent Psychiatric Research Department, Psychiatric University Hospitals Basel, Basel, Switzerland; 2 Department of Forensic Child and Adolescent Psychiatry, Psychiatric University Hospitals Basel, Basel, Switzerland; 3 Child and Adolescent Psychiatric Hospital, University of Ulm, Ulm, Germany

## Editorial

Five years after the first thematic series on “Forensic Child and Adolescent Psychiatry and Mental Health” [1] we now publish the next series on this topic. During these last years the European Association for Forensic Child and Adolescent Psychiatry, Psychology and other involved professions (EFCAP) (http://www.efcap.org/), has continued to focus on the medical and educational interests of young people in the police and justice systems. To encourage the interdisciplinary exchange between countries, international EFCAP Congresses have taken place in Amsterdam (2008), Basel (2010), Berlin (2012), Manchester (2014) and Porto (2016). Starting in 2007 with the article of Doreleijers and colleagues on “Sexual risk behavior and pregnancy in detained adolescent females” [2], Child and Adolescent Psychiatry and Mental Health (CAPMH) has published many papers that focus on adolescent forensic topics. Having this in mind, the Board of EFCAP discussed to publish thematic series of papers on an annual basis in the future. With the journal editors’ agreement, the board applauded the idea to make CAPMH their ‘official’ journal. The EFCAP Board is very grateful for this opportunity, as the open access approach of CAPMH guaranties a broad reception and distribution of juvenile forensic research that is not in the mainstream of child and adolescent psychiatric research.

To consolidate the collaboration between EFCAP and CAPMH, Cyril Boonmann has been appointed as liaison editor. He studied psychology at Leiden University and obtained his PhD at the department of child and adolescent psychiatry of the VU University Medical Center Amsterdam. Furthermore, he worked as a researcher at the Research and Documentation Centre (WODC) of the Dutch Ministry of Security and Justice. Since May 2015 Dr. Boonmann works as research psychologist at the Department of Forensic Child and Adolescent Psychiatry as well as the Department of Child and Adolescent Psychiatry of the Psychiatric University Hospitals (UPK) of the University of Basel. Between 2011 and 2015, he was one of the first EFCAP office board members. Since 2016 he is appointed as EFCAP Board Member. His research topics of interest are mental health problems, juvenile offenders, sexual offences and online sexual offending behavior.

In the current thematic series we present a collection of five papers that cover a broad spectrum of adolescent forensic research. The prevalence of mental disorders in juvenile offenders is high, figures ranging up to 80% or even more [3]. However, mental disorders in these populations often go untreated. In order to emphasize this problem, policymakers should be informed about their national prevalence rates. For this reason, Rijo and colleagues [4] examined the prevalence rates of mental disorders, including personality disorders, in a randomized sample of male juvenile offenders assessed in the Portuguese Juvenile Justice System. In addition, they compared the prevalence rates of juvenile offenders in custodial and community-based programs.

One specific subgroup of juvenile offenders are sexually offending juveniles. Research shows that childhood abuse is highly prevalent in juveniles who sexually offended (JSOs). Although childhood abuse is related to an increased risk of mental health problems, little attention has been devoted to this relationship in JSOs. Boonmann et al. [5] examined this relationship in a group of sexually offending juveniles incarcerated in Dutch juvenile detention centers. In order to distinguish sexual offending behavior from general offending behavior, JSOs were compared with a group of non-sexually offending juveniles.

Currently, researchers have become increasingly interested in female juvenile offenders, a topic that has been neglected for many years. Although many authors have studied the relationship between psychopathic traits and antisocial behavior, research on gender differences in psychopathic traits in juvenile offenders show contradicting results. To improve the understanding of psychopathy in offending girls, Lindberg et al. [6] examined psychopathic traits and psychopathy-related background variables in a Finnish nationwide sample of violently offending girls referred to a pretrial forensic psychiatric examination. The girls were also compared to their male counterparts.

In addition, Sevecke et al. [7] examined the relationship between general dimensions of personality pathology and early traumatic experiences on the one hand, and psychopathy on the other hand in both male and female detainees in Germany. Associations between physical abuse, emotional dysregulation and psychopathic traits could only be demonstrated in delinquent boys but not in delinquent girls. Remarkable is the result that a significant relationship between trauma experiences and personality pathology could be confirmed in the lifestyle and antisocial dimensions of psychopathic personality traits, but not in the core affective and interpersonal dimensions.

Finally, although decreased activity of the stress regulation systems has been reported in juvenile males with externalizing behavior problems, results in girls are less consistent. Internalizing problems, such as trauma-related symptoms, might influence this. Therefore, Babel and colleagues [8] examined the relationship of stress regulation systems’ activity and disruptive behavior in female juveniles, including trauma related symptoms as a potential mediator, in a sample of females adolescents admitted to a closed treatment institution in the Netherlands.

We are sure that these five articles are not only of interest for adolescent forensic mental health specialists but also for mental health specialists in general. Furthermore, we hope that this thematic series will stimulate researchers and clinicians to submit papers for the next article collection on Forensic Child and Adolescent Psychiatry and Mental Health that will be published in 2017.
